# Contraceptive behavior of women in India after induced abortion

**DOI:** 10.1186/s12978-024-01802-4

**Published:** 2024-05-01

**Authors:** Labhita Das, Chander Shekhar

**Affiliations:** International Institute for Population Sciences, Mumbai, Maharashtra 400088 India

**Keywords:** Abortion, Counselling, India, Post-abortion contraception

## Abstract

Putting an end to the silent pandemic of unsafe abortion is a major public health concern globally. Adoption of post-abortion contraception is documented as a significant contributor to reduce the number of unintended pregnancies and number of induced abortions. This study aimed at investigating the post abortion contraceptive behavior of Indian women exploring the determinants of post-abortion contraceptive uptake. Retrospective calendar data for 6,862 women aged 15–49 years from fifth round of National Family Health Survey (2019–2021) was used for the study. Multinomial logistic regression method was used to model the determinant factors to post-abortion contraceptive uptake. 72.6% women reported adopting no method of contraception after the abortion procedure. A total of 27.4% women adopted some method of contraception after abortion. 14% women preferred adopting short term modern methods. Women in early reproductive age group which is the most vulnerable group in experiencing unintended pregnancies are less likely to adopt any contraceptive method after abortion. Uptake of post abortion contraception is quite low in India. Effort should be taken in the direction of bringing awareness through provision of targeted contraceptive counselling after abortion.

## Background

 A safe induced abortion does not affect future fertility, with ovulation potentially resuming immediately after the procedure. The return to fertility shows no distinction between medical or surgical abortion methods [1, 2]. Additionally, many women typically resume sexual activity shortly after undergoing an abortion. Provision of abortion presents an opportunity to provide effective contraceptive options to couples eager to prevent another pregnancy. The compelling logic behind providing post-abortion contraceptives so as to avoid future unplanned pregnancies is yet to reach a wide range of population [3, 4]. The health risk related to abortion multiplies manifold when women resort to it repeatedly [5]. Induced abortion as defined by WHO as the deliberate interruption of an ongoing pregnancy by medical or surgical means differs from spontaneous abortion (miscarriage) which refers to a spontaneous loss of pregnancy before twenty weeks [6]. In India, societal norms continue to stigmatize premarital conception and motherhood so unintended pregnancy among unmarried women also is a major concern [7]. The sociocultural norms, pre-existing stigma around abortion and provider’s attitude make it extremely difficult for the women to access safe abortion and post-abortion care even after fifty years of legalization of abortion in India. The young sexually active women are exposed to various risks like unintended pregnancies, pregnancy related morbidities and unsafe abortion [7, 8]. Poor access to and use of contraception is a key contributing factor [9]. In low and middle income countries young women are more vulnerable due to socio economic inequalities related to maternal and child care [10, 11].The 2021 amendment of medical termination of pregnancy act 1971 further liberalized abortion on many grounds [12], but the abortion law itself is yet to reach many minds in India [13, 14]. According to the report of NFHS-5 India, 2.9% pregnancies ended in abortion, 47.6% among which are because of unintended pregnancies [15]. In rural India, lack of information and/or misinformation regarding temporary methods of contraception determines the contraceptive uptake of women [16].

Adoption of contraceptive method after abortion is extremely low in India. Among the women who agree to adopt any post-abortion contraceptive method tend to discontinue within a short period [17]. The most vulnerable group in accessing contraceptive methods in India are the women in the early reproductive age group [7, 18]. With the fact that women in India have very little control over their own fertility, the chances are very high that their post abortion contraceptive decisions are affected by several external factors [19]. In India, where taboos around abortion are still in place and poor contraceptive use is a major contributing factor to unintended pregnancies which eventually end in abortion [19], the knowledge about contraceptive history and intention to use post-abortion contraception is scarce. Little is known about their contraceptive behavior, including correlates and obstacles in the pathway that inhibit their access to safe post abortion contraceptive services. There are only two national-level studies in India so far to the best of authors’ knowledge, which discuss about the contraceptive uptake of the women after undergoing an abortion [17, 20]. Studies have shown that preference for contraceptive method and women’s post abortion behavior changes over time [21, 22]. Post abortion contraceptive behavior of Indian women have not been studied exhaustively for a long period of time. With growing concerns around abortion, Government of India has taken many steps including the guidelines for Comprehensive Abortion Care (CAC) to reinforce contraceptive uptake followed by abortion [23]. To better understand where India stands now in terms of contraceptive delivery and post abortion care, some updated knowledge is required which will help the policymaker to identify the areas of major concern in quality of abortion care in India.

The objective of the current study is to understand the contraceptive behavior of the women following an induced abortion, emphasizing on finding the correlates of contraceptive adoption following a voluntary termination of pregnancy in India.

## Data and methods

### Data

The analyses were carried out using the fifth round of National Family Health Survey (NFHS-5) conducted during 2019–2021 in 28 states and 8 union territories of India. NFHS is a large-scale multi-stage cross sectional survey conducted in representative sample of households throughout India. The protocol for the NFHS-5 survey with the content of all the survey questionnaires, was approved by the Institutional Ethical Review Board of International Institute for Population Sciences, the ICF Institutional Ethical Review Board and was reviewed by the U.S. Centers for Disease Control and Prevention (CDC). NFHS-5 had collected cross-sectional data for wide range of socioeconomic, demographic and health entities from 707 districts of India, which included 30,198 primary sampling units. A primary sampling unit in an urban area is census enumeration block, and a village in rural area. Prior consent was procured from all the women before participating in the survey. NFHS-5 collected month wise data explicitly identifying the key events in the reproductive history of the women within the period of 60 months prior to the interview date. Chronological sequencing of the data provided an opportunity to identify the event of terminating the pregnancy and the following contraceptive use in each calendar month. The event of the recent termination of pregnancy was recorded in the month when the pregnancy was terminated using the letter ‘M’ for miscarriage, ‘A’ for abortion and ‘S’ for stillbirth. Among the 724,115 women in the reproductive age group 2.9% pregnancies were terminated [15]. The final sample consisted of 6,862 women for the analysis who underwent abortion within the period of 60 months prior to the survey date.

### Outcome variable

Main outcome variable for the study was adoption of any contraceptive method after the event of abortion. The utilization of any contraceptive method was determined by analyzing data from a calendar matrix, which displayed month wise data for various contraceptive options. No exposure period was considered as there is evidence that earliest ovulation can occur as early as on tenth postabortal day suggesting that they should be supplied with contraceptive method within ten days after the abortion [24–26].

### Explanatory variables

The explanatory variables were main reason for abortion (unplanned pregnancy, contraceptive failure, any health related reasons and other reasons like sex of the child, other family member’s opinion, last child too young, economic reasons and foetus had congenital abnormality), method of abortion (medical method of abortion, manual vacuum aspiration, surgical methods and other unlisted methods too which may include unsafe methods of abortion), person who performed the abortion (trained healthcare personal, Ayush/Dai (AYUSH-Ayurveda, Yoga & Naturopathy, Unani, Siddha & Homeopathy and Dai are traditional birth attendant), untrained person like a relative or a friend and the woman herself) and the timing of abortion (first trimester, second trimester and third trimester). Additional sociodemographic and socioeconomic explanatory variable considered were wealth index of the household, age (estimated age at the time of abortion from the current reported age), caste (Schedule caste (SC), Schedule tribe (ST), Other backward caste (OBC is a collective term used by the Government of India to identify the educationally or socially disadvantaged castes) and other categories of caste), religion (Hindu, Muslim, Christian and Other), educational attainment (No education, primary education, Secondary education and higher), place of residence (Urban and rural) and exposure to mass media. Stata 14.2 statistical package was used to carry out all the analyses. Sampling weights available with the dataset were applied for all the analyses to account for the survey design.

### Analytical plan

The multinomial regression model was used to identify the correlates of post-abortion contraceptive adoption among the women seeking abortion services. The contraceptive methods were grouped into four categories; permanent methods, which consist of male and female sterilization cases; reversible method considering the method of intrauterine contraceptive device (IUCDs); short term modern method which included pills, condoms, foam & jelly, injectable and diaphragm and traditional method including rhythm, withdrawal and periodic abstinence. Those who did not use any method were categorized under the heading of ‘no method’. For the analysis purpose permanent methods and IUCDs have been combined as these two are long term methods as compared to the listed methods.

## Results

### Choice of contraception after abortion and sociodemographic characteristics

**Fig. 1:**
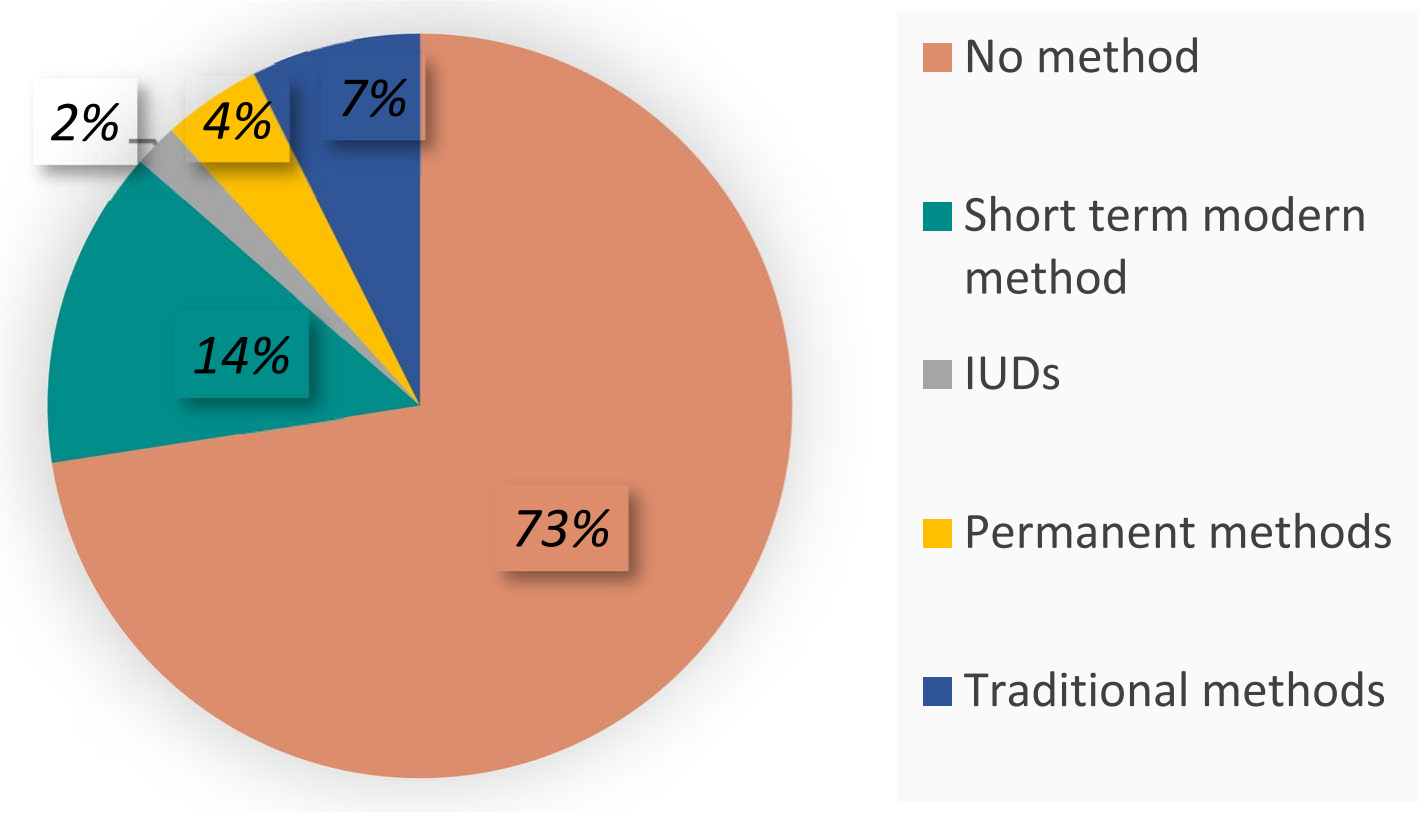
Percentage distribution of the contraceptive methods adopted by the women aged 15–49 years who underwent an abortion in the past five years NFHS 5 (2019–2021)

Among 6,862 women who underwent abortion during the last five years (sixty months) from the date of interview, only 27% of the women adopted some contraceptive method after abortion. Another 73% among the abortion seeking women did not adopt any contraceptive method. 14% of women adopted short term modern methods, 1.9% women adopted IUDs, 4.3% women they themselves or their partner underwent sterilization, 7.4% women had adopted some traditional contraceptive method after abortion (Fig. 1). Short term modern methods were observed as the highest preferred method for post abortion contraception.

A small percentage of women at the early reproductive age group (15–24 years) were observed to have adopted any contraceptive method after abortion and this was also found to be true with most of the women at late reproductive age group (more than 35 years) (Table 1). A higher proportion of women from the poor wealth quintile opted for traditional method as well (9.67%). More women from Schedule caste and OBC category underwent abortion but comparatively more women from schedule tribe category opted for traditional method of contraception (9.4%). Among all the Christian women who underwent an abortion, 84% did not adopt any contraceptive method after abortion. 11% of women who did not have any education chose traditional contraceptive methods. Usage of temporary (short term) modern methods were observed to be increasing with educational attainment among women. Nearly three –fourths of rural women were non-user of post abortion contraception. Women who had mass media exposure were observed to have opted for abortion but did not show any differentials in the adoption of contraceptive method after abortion. Women who underwent abortion because of any health-related issue mostly did not opt for any contraceptive method after the procedure (85.3%). Women who underwent abortion in the first trimester of their pregnancy only 14.5% of them opted for a short-term modern method. 90% of women aborting their pregnancy in the third trimester did not opt for any contraceptive method. Most women used medical method for abortion and only 14.7% among them preferred short term modern contraceptive methods. Only 5.1% women seeking abortion from a trained healthcare professional agreed on adopting IUDs, a long-term reversible method.

**Table 1:** Choices of post abortion contraceptive method by social and demographic characteristics, NFHS 5 (2019-2021)

Background Characteristics		Type of contraceptive method				
	Did not adopt (***N***=5,034)	Short term modern methods	IUDs	Permanent methods	Traditional methods	Total
**Age at abortion**						
15-19	86.11	10.02	0.75	0.47	2.65	519
20-24	76.80	13.14	2.08	2.49	5.50	2233
25-29	71.04	14.02	2.06	4.81	8.07	2250
30-34	63.99	17.14	1.88	7.00	9.98	1179
35+	68.22	13.15	1.60	6.53	10.51	681
**Wealth Index**						
Poor	71.17	13.15	1.17	4.84	9.67	2200
Middle	74.83	13.51	1.72	3.61	6.33	1477
Rich	72.47	14.55	2.46	4.19	6.32	3186
**Caste**						
Schedule caste	73.65	12.93	1.23	5.04	7.15	1586
Schedule tribe	74.06	10.88	1.50	4.14	9.42	405
OBC	74.73	12.74	1.52	3.53	7.49	2902
Others	68.19	16.95	3.05	4.78	7.03	1969
**Religion**						
Hindu	72.14	13.95	1.92	4.51	7.47	5,818
Muslim	74.16	14.64	1.89	2.74	6.58	763
Christian	83.95	4.97	2.19	5.24	3.66	103
Others	72.83	13.45	0.73	2.60	10.39	179
**Educational attainment**						
No education	68.91	13.91	0.93	5.03	11.22	892
Primary	69.95	12.56	1.27	6.88	9.33	766
Secondary	72.84	13.74	2.13	4.16	7.13	3,833
Higher	75.64	14.97	2.19	2.64	4.56	1,371
**Place of residence**						
Urban	69.63	14.95	3.07	4.99	7.36	2,565
Rural	74.31	13.24	1.19	3.85	7.41	4,297
**Exposure to mass media**						
No	71.83	11.73	1.45	5.32	9.67	1,193
Yes	72.72	14.33	1.98	4.05	6.91	5,669
**Reason for abortion**						
Unplanned pregnancy	64.99	17.87	2.26	4.96	9.92	3,005
Contraceptive failure	62.49	20.91	0.97	4.90	10.73	233
Health related issues	85.25	6.59	1.36	3.09	3.72	1,632
Others	74.77	13.01	1.88	4.13	6.20	1,991
**Time of abortion**						
First Trimester	72.07	14.53	1.88	4.01	7.51	5867
Second Trimester	85.15	7.72	0.87	2.28	3.97	842
Third Trimester	90.03	1.29	1.76	3.43	3.49	153
**Method of abortion**						
Medication	72.39	14.7	1.46	3.09	8.36	4,642
MVA	68.41	13.93	2.8	8.56	6.31	791
Surgical	74.93	11.02	3.19	5.64	5.23	1,221
Others	78.33	12.14	0.38	6.43	2.72	208
**Person who performed abortion**						
Trained healthcare professional	75.19	11.96	2.27	5.46	5.12	4,824
Ayush/ Dai	76.23	19.79	2.09	0	1.9	42
Untrained person	75.21	12.83	0.27	1.64	10.06	277
Self	64.68	19.29	1.07	1.48	13.48	1,719
Total	4,979	952	130	293	507	6862

### Post-abortion contraceptive uptake and associated variables

The potential association of the selected variables and post abortion contraceptive uptake was further examined controlling for sociodemographic characteristics and the variables associated with abortion using the multinomial logistic regression. The results are presented with relative risk ratios with 95% confidence intervals in Table 2. Age at the time of abortion has shown a significant association with adoption of contraceptive methods. Women in senior reproductive age group are more likely to adopt any contraceptive method as compared with women in (15–19) years (*P* < 0.001) (Table 2). Women from economically richer strata are less likely to rely on traditional methods for post abortion contraception. The relative risk ratios depicting association between post abortion contraceptive uptake and different categories of caste were insignificant. Muslim women are significantly less likely to adopt any method, especially the traditional contraceptive methods (RR: 0.77, *P* < 0.005). Women belonging to rural communities are less likely to adopt any contraceptive method compared with their urban counterparts. When the pregnancy is aborted because of any health-related issues of the women, the contraceptive uptake of any category is highly unlikely (RR: 0.47, *P* < 0.005). While comparing with first trimester abortion, women aborting their pregnancy in second trimester are less likely to adopt any contraceptive method (RR: 0.63, *P* < 0.005).

**Table 2:** Associated factors to post abortion contraceptive uptake of the women in India, NFHS 5 (2019–2021)

Background Characteristics	Relative risk ratios (*N* = 6862)			
	Adopting any method Vs no method	Permanent method & IUDs Vs no method	Short term modern methods Vs no method	Traditional methods Vs no methods
**Age at abortion**				
15–19				
20–24	1.49***(1.11 1.99)	2.44**(1.04 5.69)	1.4*(0.97 2.02)	1.28 (0.79 2.08)
25–29	1.9***(1.42 2.53)	3.94***(1.71 9.1)	1.49**(1.03 2.15)	1.98***(1.23 3.18)
30–34	2.56***(1.9 3.44)	6.01***(2.58 13.96)	1.96***(1.34 2.87)	2.43***(1.49 3.96)
35–39	2.66***(1.93 3.67)	5.12***(2.13 12.29)	2.04***(1.35 3.09)	2.93***(1.74 4.93)
40–44	1.79***(1.16 2.77)	2.72*(0.91 8.17)	0.91 (0.47 1.76)	3.23***(1.73 6.06)
45–49	1.45 (0.58 3.57)	1.89 (0.21 16.98)	0.34 (0.04 2.66)	3.45**(1.16 10.29)
**Wealth Index**				
Poorest				
Poorer	0.86 (0.72 1.04)	0.75 (0.52 1.09)	0.93 (0.72 1.2)	0.85 (0.65 1.12)
Middle	0.85*(0.69 1.03)	0.62**(0.42 0.93)	1.1 (0.85 1.44)	0.69**(0.51 0.94)
Richer	0.84 (0.68 1.04)	0.68*(0.45 1.04)	1.04 (0.78 1.38)	0.74*(0.53 1.02)
Richest	0.83 (0.66 1.05)	0.73 (0.46 1.15)	1.13 (0.83 1.54)	0.62**(0.42 0.9)
**Caste**				
Schedule tribe				
Schedule tribe	0.9 (0.73 1.12)	0.73 (0.47 1.15)	0.83 (0.63 1.11)	1.05 (0.76 1.45)
OBC	0.99 (0.85 1.15)	1.01 (0.74 1.36)	0.89 (0.73 1.09)	1.09 (0.85 1.39)
Others	1.1 (0.93 1.3)	1.08 (0.77 1.51)	1.03 (0.82 1.28)	1.16 (0.88 1.52)
**Religion**				
Hindu				
Muslim	0.77**(0.64 0.94)	0.64**(0.42 0.96)	0.97 (0.76 1.25)	0.62***(0.44 0.87)
Christian	0.91 (0.67 1.26)	1.18 (0.65 2.14)	0.88 (0.57 1.36)	0.8 (0.48 1.34)
Others	0.97 (0.75 1.26)	0.43**(0.21 0.85)	0.95 (0.67 1.34)	1.37*(0.95 1.99)
**Educational attainment**				
No education				
Primary	1.03 (0.83 1.28)	1.14 (0.76 1.7)	0.97 (0.71 1.32)	1.04 (0.75 1.45)
Secondary	0.99 (0.83 1.19)	0.84 (0.59 1.19)	1.07 (0.84 1.38)	1 (0.76 1.32)
Higher	0.81*(0.64 1.02)	0.54**(0.34 0.86)	1.07 (0.79 1.46)	0.65**(0.44 0.95)
**Place of residence**				
Urban				
Rural	0.84**(0.73 0.96)	0.67***(0.52 0.88)	0.94 (0.79 1.12)	0.83 (0.67 1.04)
**Exposure to mass media**				
No				
Yes	1.03 (0.87 1.21)	1.04 (0.75 1.45)	1.1 (0.88 1.38)	0.93 (0.73 1.19)
**Reason for abortion**				
Unplanned pregnancy				
Contraceptive failure	1.1 (0.83 1.46)	1.5 (0.91 2.47)	0.98 (0.67 1.43)	1.08 (0.69 1.7)
Health related issues	0.47***(0.39 0.55)	0.49***(0.35 0.67)	0.46***(0.37 0.58)	0.48***(0.36 0.64)
Others	0.72***(0.63 0.83)	0.83 (0.63 1.08)	0.68***(0.57 0.82)	0.74***(0.6 0.92)
**Time of abortion**				
First trimester				
Second Trimester	0.63***(0.51 0.78)	0.63**(0.42 0.94)	0.67***(0.5 0.89)	0.57***(0.39 0.83)
Third Trimester	0.67 (0.36 1.26)	1.31 (0.58 2.97)	0.24*(0.06 1)	0.65 (0.2 2.1)
**Method of abortion**				
Medication				
MVA	1.43***(1.19 1.71)	2.13***(1.57 2.9)	1.29**(1.01 1.64)	1.16 (0.85 1.59)
Surgical	1.13 (0.96 1.33)	1.72***(1.3 2.27)	0.95 (0.76 1.19)	1.01 (0.77 1.33)
Others	0.65**(0.43 0.99)	1.12 (0.58 2.14)	0.45**(0.22 0.89)	0.65 (0.32 1.31)
**Person who performed abortion**				
Trained healthcare professional				
Ayush/ Dai	1.3 (0.67 2.53)	0.94 (0.22 4.02)	1.76 (0.79 3.92)	1.01 (0.3 3.38)
Untrained person	0.98 (0.73 1.32)	0.3***(0.12 0.74)	1.11 (0.76 1.64)	1.3 (0.83 2.02)
Self	1.55***(1.35 1.78)	0.45***(0.31 0.64)	1.77***(1.48 2.12)	1.99***(1.6 2.47)
**Constant**	0.290	0.049	0.123	0.098

Significance level *p < 0.1. **p < 0.05. ***p < 0.01.

When the abortion is carried out using Manual Vacuum Aspiration (MVA), there are higher chances of adopting any contraceptive method (RR: 1.43, *P* < 0.005). The abortion carried out by the women herself using medical method of abortion are more likely to adopt short term modern methods and traditional methods but are less likely to adopt any permanent or long-acting reversible method (*P* < 0.005).

## Discussion

In an endeavor to explore the contraceptive practices of women following induced abortion, utilizing a nationally representative dataset, the study has uncovered several critical findings. The uptake of any contraceptive method after induced abortion was notably low in India, particularly among women in early reproductive age groups. Despite concerted efforts by the government and concerned agencies, India continues to struggle in making significant advancements in post-abortion contraceptive uptake. Among the younger women who had chosen to adopt some method, a very small proportion adopted effective method of contraception after undergoing an induced abortion. In the resource poor setting, proportion of women receiving effective post abortion contraceptive method generally found to be low [27], similar scenario might be happening in India, unavailability of different choices for family planning at the healthcare facilities could be an impeding factor in higher use of post-abortion contraceptive method. A study in Ghana reported that the girls and women using unsafe abortion method were more likely to adopt contraceptive method when compared with those had a safe abortion. By induction, the vulnerability of the young women that underpins unsafe abortion had translated into contraception use [28]. This might also stem from their negative experiences with the health repercussions following unsafe abortions. Sex education and heightened awareness about sexual health would indeed play a pivotal role in enhancing post-abortion contraceptive utilization.

The analysis unveiled a preference among women for short-term contraception methods over intrauterine devices (IUDs) as reversible options. A significant proportion of women were noted to continue relying on traditional methods even after undergoing induced abortion. One potential explanation could be a lack of awareness regarding the array of contraception options available, particularly in relation to short-term, temporary modern methods [20, 22]. The study further validates that the uptake of post-abortion contraception is significantly influenced by both the method utilized during the abortion procedure and the individual conducting the abortion. This finding can be attributed to women gaining exposure to different contraceptive options when seeking abortion services at healthcare facilities. Additionally, the provision of post-abortion counseling or simple advice and guidance on contraceptive usage by healthcare providers significantly impacts women’s adoption of contraception [29]. Women residing in rural areas were found to be lagging behind in terms of post abortion contraceptive uptake. A probable explanation could be that awareness has not yet penetrated the remote locations of India, also myths around contraceptive use are additional barriers to its uptake. Women from rural areas are disadvantaged in terms of access to post-abortion contraception [30]. Uptake of post-abortion contraceptive method is hence poor in rural areas [31]. However one must be cautioned that evidence also found coercion in such circumstance of higher use of any method due to provider’s bias or attitude especially in rural areas [32].

The association between time of induced abortion with the post abortion contraceptive uptake is also apparent from the study. Time of abortion itself is dependent on various factors like partner’s support, economic status and societal factors which in turn plays critical role in the decision-making process of the women about using contraceptive method after seeking an abortion [33]. The highest probability of contraception adoption is confined to the women aborting their pregnancies in the first trimester. Early detection of unwanted pregnancies and accessing safe abortion care to the women can encourage the uptake of contraceptive methods. It was also evident from the study that women who underwent abortion with Manual Vacuum Aspiration (MVA) are more likely to adopt any contraceptive method. This could be a result of exposure of the women to information and a wider basket of options available on contraceptive methods at the health care facilities. Such exposure tends to motivate the women to adopt a contraceptive method.

Rather than merely introducing family planning methods more qualified counselling techniques should be employed which would explicitly focus on preventing subsequent termination. In order to increase awareness of post-abortion family planning methods among remote and socially disadvantaged segments of society, it is imperative for the local health workforce to adhere to the guidelines for comprehensive abortion care. Since it was found that the motivation of women to adopt contraception is highest immediately after abortion [34], it was also established by another study that the women receiving facility-based family planning services had fewer subsequent unplanned pregnancies [35]. A clear indication from this study finding is that there’s a profound need of mandatory and effective counselling to the women undergoing abortion with a strong emphasis on adopting contraceptive methods. Counselling targeted population would escalate the reach among the vulnerable groups of women. Policies and programs related to comprehensive post abortion care in India should emphasize on outreach of the services to the intended pockets of the population. This article explicitly concludes that there is a critical need for a substantial increase in post-abortion contraceptive utilization to prevent unintended pregnancies and to mitigate subsequent consequences to women’s health.

The strengths of this study lie in its utilization of a nationally representative sample, ensuring the reliability and applicability of the findings. The research delved into the contraceptive practices of women who experienced an abortion, a group often facing physical and emotional vulnerabilities, as well as the risk of repeat pregnancies that could detrimentally impact women’s health.

The present study had certain limitations. Firstly, the data utilized was obtained from a cross-sectional survey, which may be susceptible to recall bias. Additionally, the information regarding abortion could only be obtained for the most recent pregnancy in the calendar data, thus preventing the examination of contraceptive behavior prior to this recent pregnancy.

## Conclusion

The majority of women who had an induced abortion did not utilize any contraceptive method. Furthermore, among those who did report using a method, a considerable number resorted to traditional methods. Consequently, the study suggests a need to enhance post-abortion contraceptive services especially after an induced abortion. India’s Family Planning 2030 initiative, introduced in 2022, aims to promote healthy timing and spacing of pregnancies by improving access to information and services, particularly targeting young and vulnerable populations. Additionally, the Comprehensive Abortion Care Guidelines of 2018 recommend post-abortion contraceptive counseling and encourage the voluntary adoption of intrauterine contraceptive devices (IUCD) and other modern methods. Effective implementation of these interventions is crucial to realize the broader objectives of achieving Sustainable Development Goals 3 and 5 within India’s specified timeframe.

## Data Availability

Publicly available datasets were analyzed in this study. This data can be found here: https://dhsprogram.com/data/available-datasets.cfm.
